# Supplementary material for: Are carbon emissions trading and green financial instruments synergistic? -Comprehensive quantitative research based on content analysis
Source: PLoS One. 2024 Mar 7;19(3):e0298601. doi: 10.1371/journal.pone.0298601 (PMC10919601; doi:10.1371/journal.pone.0298601)
Supplement: S1 Data — (ZIP) [file pone.0298601.s001.zip › Data/地级市Z_F工作报告.zip!/╕ú╜¿/─╧╞╜╩╨/└┤╫╘╣1⁄2╓┌║┼í╛┬φ┐╦╩2╛▌═°í┐_2010.docx]

各位代表：

现在，我代表南平市人民政府向大会报告工作，请予审议，并请市政协委员和其他列席人员提出意见。

一、抢抓机遇，奋力拼搏，海西绿色腹地建设取得新成效

2009年，在省委、省政府和市委的正确领导下，在市人大、市政协的监督支持下，我市各级政府认真贯彻党的十七大、十七届三中、四中全会精神，深入学习实践科学发展观，全面落实国务院《关于支持福建省加快建设海峡西岸经济区的若干意见》和省委、省政府《实施意见》，团结全市人民抢抓机遇，树立信心，锐意进取，经济社会发展取得新成效。

（一）积极应对金融危机，发展态势持续向好。坚决实施中央和省上应对国际金融危机的一系列政策措施，全力保增长、保民生、保稳定，基本实现市三届人大四次会议确定的年度目标。全市生产总值621.74亿元，增长13.9%，增幅居全省第三。全市财政总收入52.26亿元，增长9.75%；地方级财政收入31.39亿元，增长11.91%。市本级财政总收入15.29亿元，增长15.70%，完成年度预算104.91%；地方级财政收入7.34亿元，增长11.63%，完成年度预算101.48%。规模以上工业产值597.07亿元，增长20.2%；农林牧渔业产值229.02亿元，增长6.3%。全社会固定资产投资502.03亿元，增长26.7%。实际利用外资（验资口径）6167万美元，增长5.3%；外贸出口6.45亿美元，增长1.8%。社会消费品零售总额244.98亿元，增长16.4%。旅游接待总人数1186.09万人，旅游收入137.37亿元，分别增长15%和20%。城镇居民人均可支配收入15867元，实际增长10.4%；农民人均纯收入6116元，实际增长10.2%。城镇登记失业率3.52%。年末金融各项存款余额626.71亿元，增长24.79%；贷款余额493.82亿元，增长26.42%。万元GDP综合能耗下降4.2%，二氧化硫减排5.1%，化学需氧量减排1.12%。

（二）主动融入海西建设大局，绿色腹地地位和作用逐步凸显。围绕海西建设“我国重要的自然和文化旅游中心”的功能定位，编制旅游发展总体规划，实施一批重大旅游基础设施项目，为构建大武夷旅游经济圈打好基础；围绕建设闽浙赣三省交界新兴中心城市，完成闽北新兴发展区域战略规划编制，着手编制总体规划，做好区域保护和控制工作，武夷新区纳入省委、省政府海西建设的重大战略布局；围绕构建现代立体交通体系，一批高速公路和高速铁路相继开工建设，武夷山机场扩容和新机场规划选址前期工作加快推进，闽浙赣交界重要交通枢纽的布局基本形成。

（三）全面推进基础设施建设，发展条件进一步改善。武邵、宁武、龙浦、松建四条高速公路同时在建，总里程430公里；邵光、南平绕城、建闽、延顺、顺邵高速公路前期工作加快推进。京福高速铁路南平段200公里开工，峰福铁路电气化南平段改造工程竣工，南三龙、浦建龙梅、衢宁铁路前期工作加快；向莆铁路南平连接线工程进展顺利，南平东支线铁路改建方案已经确定。首座500千伏变电站投入运行，闽北电网实现从220千伏向500千伏网架提升；光泽凯圣生物质热电厂建成投产；邵武火电厂改扩建项目“初可”报告通过省内专家评审。加强政银战略合作，获得银行贷款授信额度148亿元。

（四）坚持突出突破工业，产业结构逐步优化。全市产值超亿元企业110家，比上年增加20家，其中超5亿元企业19家，比上年增加7家。二产比重提高近1个百分点，三次产业结构为22.5：40.2：37.3。南纸六号机组、南铝工业用铝复合型材技改等37个重点项目建成投产，太阳电缆500千伏超高压立塔交联电缆等一批项目加快建设。新增中国驰名商标3件、福建省著名商标40件、福建名牌产品27个。太阳电缆、圣农发展同时在深交所上市，募集资金15.08亿元。闽北经济开发区、南平工业园区和荣华山产业组团建设初具规模，全年基建投资12.77亿元，建成标准厂房25.74万平方米，引进企业57家。县（市、区）工业平台加快拓展，一批工业项目入驻。着力培育旅游养生、生物、创意等特色产业。按照建设国际性旅游度假城市要求，推进茶博馆、印象大红袍山水实景演出等一批项目建设，成功举办第十二届海峡西岸武夷国际投资洽谈会和国际山水茶体育·旅游节、首届海峡两岸大圣文化旅游节，武夷山成为海峡两岸茶业博览会固定举办地，政和佛子山和顺昌宝山被评定为国家级风景名胜区，浦城匡山获批国家级森林公园，建阳大历溪森林狩猎项目获得批准。积极参与“6·18”、“9·8”等重大经贸活动，先后在泉州、杭州、广东、香港、台湾等地举办招商活动，签约项目63个，总投资93亿元。南平农村商业银行、延州小额贷款公司获得省上批准。积极搭建银企对接平台，推进担保体系建设。

（五）推动城市化先行，城市形象明显改观。完成南平中心城市安济新城概念规划和黄墩片区、大家厂片区、后谷组团控制性规划等编制工作。滨江路改造工程、朱熹路建设工程和沿江亲水平台一期工程顺利完成，狮子山隧道打通，西城大桥建成，常坑大桥、剑州大桥、黄墩大桥、闽江大桥、闽江路、滨江东路一期工程加快建设。完成中心城区LED夜景三期工程和主干道人行道铺设等配套工程，实施玉屏山、九峰山公园改造工程，全面推行环卫保洁市场化运作，城市管理得到加强。各县（市、区）启动一批基础性、功能性和产业性城建项目，邵武市大力实施城市亮化、美化、彩化、净化工程，建阳市建成嘉禾大道、宋慈广场和滨江大道亲水景观平台，浦城县加快推进梦笔大道、兴华北路、城东路建设，武夷山市完成武夷大道改扩建工程，建瓯、顺昌、松溪、光泽、政和等县（市）加快推进城市建设。建瓯、浦城污水处理厂投入试运行，光泽、松溪、政和污水处理厂完成主体工程，顺昌污水处理厂开工建设。

（六）加快中心村镇建设，农业农村发展基础不断夯实。全市先行规划建设的22个中心镇、313个中心村，已有19个镇、249个村完成规划，179个中心村启动建设，10510户农民按规划建房。规模农产品加工企业648家、新增18家，农民合作经济组织1433家、新增223家，农村信息服务站点919个、新增334个。农业“五新”推广100项，农作物良种覆盖率96%。完成土地开发整理复垦4万亩，新增耕地5200亩；实施烟基项目受益农田14.7万亩，中低产田改造、标准化农田建设、高标准鱼塘改造4.45万亩。完成农村公路路网工程600公里。水库除险加固14座，治理水土流失20.2万亩。新增农村饮水安全人口13.1万人。97%的乡镇建成简易垃圾处理场，70%的建制村实现垃圾简易处理。新建沼气池9500口。全市商品林登记发证面积1889.9万亩，96.7%的集体商品林完成登记发证，97.1%的建制村完成生态公益林管护机制改革。召开深化推进“南平机制”工作会议并举办十周年成就展，持续推进农村工作机制创新。

（七）注重统筹协调，社会事业健康发展。认真落实教育优先发展战略，实施中小学合格校建设、农村中小学现代远程教育工程，新建农村寄宿制学校宿舍和食堂40座。武夷山市通过省“双高普九”评估验收。南平四中恢复办学，南平高级中学通过省一级达标校评估验收。闽北职业技术学院完成搬迁，福建林业职业技术学院江南校区投入使用。支持武夷学院提高办学水平，恢复福建农林大学南平校区办学。加强教师队伍建设，稳步实施中小学教师绩效工资制度改革。举办南平市第二届科技成果交易会，签约项目54个，总投资7.8亿元。可持续发展实验区通过省上验收。公共卫生服务加强，甲型H1N1流感有效防控。加强社区卫生服务中心建设，南平市人民医院被评定为三级乙等中医院。乡镇卫生院公益性运行机制改革顺利推进，实行人员工资财政统一支付。完成20户以上自然村广播电视村村通建设和8个高山台无线覆盖工程，82%的建制村实现广播电视光缆联网，建成有线数字电视整体转换市级平台。成功举办南平市第二届艺术节、武夷山全国脚斗士精英赛、环世界遗产地——武夷山旅游景区公路自行车赛、邵武全国汽车拉力锦标赛等活动。持续稳定低生育水平，人口自然增长率为6‰，80%的县（市、区）成为国家、省计生优质服务县。武夷山、光泽、邵武、建阳四个县（市）被认定为原中央苏区县。积极开展群众性精神文明创建活动，开展全国、省道德模范人物推荐和第二届“感动闽北十大人物”评选活动，6个单位、5个村镇被评为全国文明先进单位（村、镇）。妇女、儿童、老龄和残疾人工作得到加强，社会福利和慈善事业稳步发展。援建彭州市九尺镇工作进展顺利。民族宗教、防灾减灾、档案方志、老区库区等工作取得新成效。

（八）加强社会建设，民生水平持续提高。完成18项为民办实事项目。全年新增城镇就业人员2.48万人，农村劳动力转移就业5.39万人。基本医疗保障制度覆盖城乡居民，新农合人均筹资额从90元提高到100元，参合率96.33%，城镇职工和居民参保人数完成省定任务。实施廉租住房项目15个、2406套、11.70万平方米，已竣工908套、4.16万平方米，发放廉租住房租赁补贴348.47万元。实施“造福工程”搬迁1万人。发放家电下乡补贴3019万元。深化“平安南平”建设，开展优化发展环境治安专项整治活动，妥善化解各类矛盾纠纷，群众对社会治安满意率96.31%。深入开展重点行业和领域的专项整治，建立医患纠纷调解处理机制，强化食品药品安全管理，安全生产形势总体平稳。国家级生态示范区创建通过验收，积极开展生态市建设，推进节能减排工作，实施闽江流域水环境综合整治，加强饮用水源保护，禁建区内规模养殖场治理率达100%。国防动员、民兵预备役和双拥共建等工作有新进展。

（九）坚持依法行政，政府自身建设得到加强。深入开展学习实践科学发展观活动，引导广大干部解放思想、转变观念、创新思维、增强信心，提高经营运作和公共服务水平，提升推动科学发展的能力。启动事业单位改革，重新核定编制，累计核减编制3000多个。推进审批制度改革，完善行政服务中心，成立招投标服务中心，整合筹建信息中心，推行政府信息公开。加强机关效能建设，规范行政自由裁量权。自觉接受人大法律监督、工作监督和政协民主监督，办理市人大代表议案和建议119件、市政协委员提案239件，办复率均为100%。密切与民主党派、工商联、人民团体和无党派人士的联系，主动听取并积极采纳社会各界人士的意见和建议。坚持政府部门述职述廉制度，严格落实廉洁自律各项规定，查处政府系统违纪违法案件135件、135人，反腐倡廉工作不断加强。

成绩来之不易，这是省委、省政府和市委正确领导的结果，是市人大和市政协监督支持的结果，是历届政府积累积淀的结果，是全市人民共克时艰的结果。在此，我代表南平市人民政府，向全市人民，向全体人大代表、政协委员，向各民主党派、工商联、人民团体、无党派人士和离退休老同志，向中央、省直驻南平机构和驻军、武警、公安干警，向包括港澳台同胞、海外侨胞、国际友人在内的所有关心、支持闽北发展的社会各界人士，致以崇高的敬意和衷心的感谢！

在看到成绩的同时，我们也清醒认识到存在的问题。主要体现在：闯拼精神不足。有些地方、部门思想不够解放，争先意识不强，创新不足，思路不活，办法不多。主观上自我减压，目标上留有余地，标准上降格以求，工作中因循迁就。发展相对滞后。经济总量小，二产比重偏低。产业基础作用发挥不够，重点骨干企业规模偏小。引进的生产性项目还比较少、不够大。基础条件较差。虽然一批重大交通项目相继开工，投资力度前所未有，但突破交通瓶颈还需要一个过程。部分区域电网网架较为薄弱，电源支撑点不足。统筹能力有限。社会事业欠账较多，优质资源少。社会保障标准较低，就业结构性矛盾突出，公共服务还不能满足群众需求。发展环境有待改善。政府职能需要进一步转变，一些单位和工作人员责任意识不强、执行力不强，存在“小吏大权”甚至不作为、乱作为等失职、渎职现象。安全生产仍有隐患，社会不稳定因素仍然存在。我们要高度重视这些问题，采取有力措施，切实加以解决。

二、全力攻坚，突破重点，有效推进2010年工作

2010年，是本届政府实施“十一五”规划的最后一年，也是全面落实国务院《若干意见》、加快推进海西绿色腹地建设的关键一年。政府工作的总体要求是：全面贯彻落实党的十七大和十七届三中、四中全会精神，以邓小平理论、“三个代表”重要思想和胡锦涛总书记在福建考察时的重要讲话精神为指导，深入贯彻落实科学发展观，按照中央“五个更加注重”要求和建设海峡西岸经济区的战略部署，紧紧抓住发展第一要务，坚持“五个精心谋划”、“六个求突破”，在扩大内需中保增长，在强化支撑中促转变，在改革开放中激活力，在改善民生中促和谐，全面实现“十一五”目标，科学编制“十二五”规划，加快推进海西绿色腹地建设，持续推进南平经济社会又好又快发展。

全市经济社会发展的主要预期目标是：生产总值增长13.5%；规模以上工业产值增长20%；农林牧渔业产值增长6%；财政总收入和地方级财政增长12.5%，其中市本级财政总收入和地方级财政收入增长12.5%；全社会固定资产投资增长20%；实际利用外资（验资口径）增长10%，外贸出口总额增长8%；城镇登记失业率控制在4%以内；社会消费品零售总额增长15%；旅游接待人数和收入分别增长11%和13.5%；人口自然增长率控制在7‰以内；城镇居民人均可支配收入实际增长8%，农民人均纯收入实际增长8%；万元生产总值综合能耗下降3.47%，二氧化硫排放量控制在3.18万吨以内，化学需氧量排放量控制在4.37万吨以内。在实际执行中，力求更快更好一些。

确定上述发展目标，是实现“十一五”规划的必然要求，也是加快海西绿色腹地建设的客观需要，更是基于对发展形势的科学判断。今年是经济形势最为复杂的一年，但总体上宏观经济发展环境将逐步好转，中央支持海西发展，国家和省上出台一系列扩大内需的政策，我市一批重大基础设施相继开工和建成，闽北加快发展迎来了重大历史性机遇。我们要正确把握形势，抢抓发展机遇，转变发展方式，树立绿色理念，发展绿色经济，打造绿色城市。举全市之力，致力于做足“通”的文章、以现代立体交通引领发展，致力于推动产业振兴、加快转变经济发展方式，致力于大力度推进城市建设、增强城市集聚功能，致力于加快中心村镇建设、夯实农业农村发展基础，致力于教育优先发展、全面提升教育水平和全民素质，加快推动闽北科学发展、跃升发展。

（一）打好现代立体交通攻坚战，融入发达地区经济半径

加快现代立体交通体系建设。建成武邵高速公路和武夷山机场高速公路118公里，全市高速公路通车里程达440公里。加快建设宁武、龙浦、松建高速公路344公里，开工建设建闽、邵光高速公路125公里，做好南平绕城、延顺、顺邵高速公路前期工作。完成国、省道干线公路网改造120公里，继续完善农村公路网络。加快建设京福高速铁路南平段200公里和向莆铁路南平连接线工程，开工建设南三龙铁路南平段33公里，改造南平城区铁路东支线工程，积极推进浦建龙梅、衢宁和城区铁路改造前期工作。加快武夷山机场扩容改造，完善海峡两岸空中客运直航的条件，构建厦门至武夷山空中通道，争取武夷山航空口岸对台直航入境人员落地签注。推进南平“无水港”建设。加快武夷山机场新建项目前期工作，积极争取列入国家或省上“十二五”规划。

强化电力能源保障。加快建设220千伏主干网，完成建瓯东安、武夷山南源变电站扩建，开工建设光泽变电站，推进顺昌坊上、延平夏道、政和变电站等前期工作。完善电网配套设施，构筑以500千伏变电站为中心、辐射县（市、区）的220千伏网状结构，力争实现每个县（市、区）110千伏电网双电源供电。积极推进闽北第二个500千伏输变电项目前期工作。加快推进邵武火电厂改扩建项目前期工作，力争上报国家发改委核准。积极推进南平核电项目前期工作，为“十二五”规划期间动工创造条件。

优化金融环境。加大政银企战略合作力度，拓展与国家开发银行、农业发展银行和商业银行等金融机构战略合作，鼓励引导商业银行、大企业集团和战略投资者参与组团开发等经营运作。支持符合条件的企业通过上市或发行债券等形式融资，力争年内完成市高速公路公司企业发债工作。健全中小企业信用担保体系。支持建瓯石狮村镇银行加快发展，扩大村镇银行试点面，成立南平农村商业银行，实现延州小额贷款公司正式运营。

实施项目带动。把握国家政策导向，精心策划一批大项目、好项目，争取更多新增中央投资和省上投资。今年全市计划安排重点项目221个，比上年增加36个，其中在建重点项目153个，年度计划投资173.8亿元，比上年增长43.3%。落实重点项目责任制，加强征地拆迁、项目报批、落地建设、竣工投产等各个环节的协调服务，加快推进土地收储筹措资本金工作。提前做好拟建项目前期工作，促进更多项目开工建设，发挥投资拉动作用。

（二）打好产业振兴攻坚战，加快转变经济发展方式

整合提升重点产业。组织实施机械（装备）制造、纺织服装、食品加工、林产加工、冶金建材等五大产业调整振兴方案，落实扶持新“五南”式企业发展政策，对重点企业提供个性化服务，把有限资源和生产要素向领军企业集聚。实施100个重点产业项目建设，确保南纸2、3、4号机组技改项目、南铝铝业高品质铝合金圆棒、太阳电缆特种电缆、圣农产业化和兴华啤酒等一批项目建成投产，促进现有企业产能扩张。加快推进企业改制上市工作，做大做优现有品牌。鼓励企业提升自主创新能力，积极参与国家标准、行业标准制定，建设一批国家、省、市级企业技术中心。

积极培育新兴产业。充分发挥生态资源优势，培育发展旅游养生、生物、创意等特色产业。加快旅游养生产业规划编制，规划建设大型旅游休闲养生项目。积极争创国家级生物产业基地，推进海峡生物工程学院、海峡生物科技研究院、海峡生物科技产业园建设。规划建设武夷山创意产业园，重点策划推出动漫创意、旅游创意、养生创意、文化创意等项目。主动对接国家和省上培育壮大战略性新兴产业的发展规划，培育发展新能源、节能环保、新材料、光电信息等产业。

加快构建大武夷旅游经济圈。发挥武夷山“双世遗”品牌优势，集聚闽浙赣三省旅游要素，整合闽北各县（市、区）旅游资源，打造一批国家4A级旅游景区，丰富大武夷旅游产品，推进旅游交通、线路和服务对接。按照国际性旅游度假城市要求，抓好武夷山主景区和度假区的景观设计和功能完善，加快建设具有国际旅游度假接待能力的星级酒店、能承办国际A级体育赛事的运动场馆以及能举办国际大型展会的会展中心、艺术中心和自然文化遗产博物馆，实施茶博园二期、茶博馆、大红袍山庄等一批项目，推出“印象大红袍”山水实景公演。增加武夷山至厦门的航班密度，实现每两小时一航班。突出“海峡旅游”主题，积极争取对台旅游合作先行先试政策。扶持发展武夷山会展业。

做大做强工业园区。坚持集中集约集控，加快推动闽北经济开发区、南平工业园区和荣华山产业组团及各县（市、区）工业平台扩区升级，力争每个县（市、区）都有一个省级以上经济开发区。有针对性地开展择业、招商、选资活动，积极对接央企、省企、台资企业和大型民营企业，主动承接产业转移，引进规模工业企业350个，培育新“五南”式企业8—10家。积极创造条件，加快推进省级工业园区污水处理项目建设。做好外经贸工作，推动对外开放和对台交流合作纵深拓展，精心组织“5·13”投洽会和海峡两岸茶业博览会暨武夷山国际山水茶体育旅游节，使之成为闽北招商选资的重要平台和对外开放的重要窗口。

大力发展现代服务业。落实省上扶持现代物流业发展的优惠政策，规划建设一批现代物流园区和综合性物流中心，进一步健全城市商业服务网络和农村现代流通网络。加快建设南平交通运输服务中心、东岭商贸走廊，培育一批物流骨干企业和专业市场。继续抓好“商贸六项工程”和“信福工程”建设，培育商贸中心区，扶持特色专业市场，发展新型乡（镇）商业区和农副产品集散地，建设新型社区商业服务中心。落实“家电下乡”、“汽车下乡”、建材下乡和农机具购置补贴等政策，扩大消费对经济增长的拉动作用。

（三）打好城市建设攻坚战，不断增强集聚能力

精心建设南平中心城市。围绕建设组团式山水园林城市目标，拉开城市框架，经营城市产业，完善城市功能，强化城市管理。完成新一轮城市总体规划修编。加快推进江南新城、安济新城、黄墩、大家厂等片区开发建设。加大沿江快速通道建设力度，建成剑州大桥、常坑大桥、黄墩大桥、闽江大桥、闽江路和高速公路南平北互通连接线，加快沿江快速通道二期工程建设。完成玉屏山、九峰山公园改造工程。完成南平“二水源”主体工程。

规划建设闽北新兴中心城市。按照《福建省建设海峡西岸经济区纲要（修编）》的要求，积极推进南平中心城市向建瓯、建阳、武夷山、邵武一带拓展，加快谋划建设武夷新区，建设成为海峡西岸承接产业转移的制造业基地，国际性旅游观光休闲基地，闽浙赣交界重要交通枢纽和中心城市。全面开展总体规划、启动区的控制性详规和修建性详规编制工作，统筹基础设施、产业发展和城市规划总体布局。组建管理机构，有效做好区域的保护与控制，积极争取先行先试政策，加快规划建设新兴中心城市交通枢纽等一批重大基础设施项目。

加快建设县域中心城市。结合高速铁路和高速公路连接线工程、国道和省道改造工程以及防洪堤工程建设，做好新一轮城市总体规划修编，实现现代立体交通与城市建设、园区开发的有效对接。策划实施一批进城景观通道、滨江大道、城市中心广场、城市标志性桥梁、污水处理厂和垃圾发电厂等基础性项目，一批达标学校、医院、福利中心、体育场馆、文化艺术和青少年活动中心、城市景观工程、高标准社区、经济适用房小区等功能性项目，一批物流中心、商业场所、星级酒店、旅游景点、产业组团等产业性项目，形成优质资源，增强集聚能力。加快建设建溪二期、富屯溪二期防洪工程，抓紧做好建溪三期、四期及富屯溪三期、江南新区防洪工程的前期工作。加快延平、邵武、建阳、建瓯、武夷山、浦城污水处理厂管网配套建设，提高污水处理率；建成顺昌、政和、松溪、光泽污水处理厂和武夷山第二污水处理厂，做好管网配套建设。筹划建设南平江南新区污水处理厂。开工建设南平中心城市和建阳城市联盟垃圾焚烧发电厂。

（四）打好中心村镇建设攻坚战，扎实推进新农村建设

全面展开中心村镇建设。推进中心村镇建设与旧村改造有机结合，与“造福工程”捆绑运作，发挥农民主体作用，确保先行规划建设的313个中心村、22个中心镇试点取得实效。启动兴田、将口等小城镇综合体试点建设，争取每个县（市、区）都有一个试点，把中心镇建成“规划先行、功能齐备、设施完善、生活便利、环境优美、保障一体”的宜居城镇综合体。推进“耕地向规模经营集中、企业向工业园区集中、民宅向中心村镇集中、人口向城市和中心村镇集中”，做好土地“整理、置换、流转、经营”文章，整合项目、资金、资源，加快完善中心村镇基础设施，提升交通、信息、科技、教育、医疗、文体等公共服务水平。

积极发展现代农业。落实扶持粮食生产的各项政策措施，稳定粮食生产。突出“一县一特色、一乡一主业”，大力发展笋竹、茶叶、果蔬、烟叶、油茶、食用菌等种植业，培育发展畜禽、水产等现代养殖业，推动优势特色农产品规模化、组织化、标准化、机械化生产，加快推进农业产业化经营，壮大特色经济规模。扶持培育一批规模农业产业化龙头企业，推进农产品精深加工。规范发展农民专业合作社，推广“龙头企业+合作社+基地带农户”发展模式。加强对台农业合作交流，启动海峡两岸（南平）现代农业合作示范区建设，抓紧建设武夷山五夫、建瓯小松、延平王台综合园及邵武大埠岗台湾果蔬等专业园。积极组织申报国家级现代农业示范区、国家农业科技园区建设项目。实施农业“五新”推广示范工程。加强农产品质量安全和动植物疫病防控工作。依托农业资源优势，扶持培育区域特色农业品牌。

改善农业基础设施。加强农田基本建设，实施农业综合开发项目8万亩、烟田基础设施建设14万亩、土地开发整理复垦12万亩，新增耕地1.8万亩。人工造林更新51万亩，建设林业“十大基地”30万亩，修建竹山机耕路1000公里。抓好36座中小型水库除险加固工作，加快光泽茶富等水库建设前期工作。强化小流域综合治理，治理水土流失18万亩。

深化农村改革创新。稳定和完善农村基本经营制度，稳妥推进土地承包经营权流转，发展适度规模经营。深化集体林权制度改革，全面落实发证工作，落实生态公益林管护制度。培育发展林业合作经济组织。改善农村信贷服务，发展农村保险事业。持续深化“南平机制”，完善农村科技特派员制度，提升农村“六大员”的综合素质和服务能力，开展新一轮“挂村服务、互动联动”工作，进一步提高农村公共服务水平。

（五）打好教育优先发展攻坚战，促进经济社会协调发展

坚持教育优先发展。贯彻教育优先发展决定，落实教育各项补助政策，推进城乡中小学标准化建设，改造提升一批薄弱学校，促进教育公平均衡发展。加强教师队伍建设，推进教师补充机制规范化。积极推进“双高普九”，新建江南新区、新城中心、官沙田三所小学，建瓯市要确保通过省级验收，浦城县、光泽县要通过省级指导性评估。加大高中阶段教育普及力度，推进高中学校达标升级创建工作，积极推进顺昌一中、建瓯一中、武夷山一中、松溪一中创建省一级达标学校。完成南平四中校园改造，加快建设南平一中江南校区。重点发展职业技术教育，整合南平中心城市中等职业教育资源，加快建设南平职业中专学校、闽北卫校新校区和南平农校职教中心，支持武夷山旅游职业学院建设，加强闽北职业技术学院建设。扶持武夷学院四期扩建和桃源现代信息服务学院，提高武夷学院办学质量。支持福建农林大学南平校区扩大办学。重视发展学前教育、继续教育和老年教育，积极发展特殊教育和少数民族教育，全面推进素质教育。进一步集聚和吸引高层次人才，优化人才队伍结构，强化人才支撑。

发展各项社会事业。积极推进科技进步，举办科技成果交易会，完善农村科技服务体系，提高企业技术创新水平，加强知识产权保护。加强农村医疗机构基础设施建设、社区卫生服务机构标准化建设。推进市、县两级各类医院达标升级工作，实现南平市第一医院创“三甲”目标，加快省立医院武夷山分院、邵武医院等病房大楼建设。加强医疗卫生队伍建设，提升公共医疗服务水平。办好第三届朱子文化节、四省四市民间艺术节、第四届农村艺术节等重大文化活动。加快数字电视工程建设。广泛开展群众性体育活动，全力备战第十四届省运会。做好第六次全国人口普查工作。推进人口计生综合改革试点，稳定低生育水平，提高人口素质，优化人口结构，实现创先进目标。

加强就业和社会保障。多渠道扩大就业途径，新增城镇就业2.3万人，农村劳动力转移就业5.2万人。鼓励高校毕业生到基层、中小企业就业，支持劳动者自主创业和自谋职业。加强职业技能培训，实行就业困难人员和零就业家庭援助。完善城乡社会保障制度，将财政支出和转移支付更多地用于社会公共保障体系建设。实行城镇职工基本医疗保险设区市统筹，实现“一卡通”服务。推进事业单位养老保险制度改革，做好农民工社会养老保险异地转移工作，扩大社保覆盖面。做好被征地农民社会保障工作。加强城乡低保规范化管理，做到科学定标、按标施保、应保尽保、动态管理。完善城乡医疗救助制度。加大对原中央苏区、革命老区、贫困地区、少数民族村、边远地区和库区的扶持力度，改善欠发达地区生产生活条件。发展慈善、社会福利和残疾人事业，加强老龄工作，切实维护和保障妇女、儿童合法权益。

办好惠民利民实事。教育卫生方面，实施中小学校舍安全工程项目50个，建筑面积8万平方米；巩固和发展城镇居民基本医疗保险，完善新型农村合作医疗制度，巩固参合率，提高受益率；实施城乡公共卫生服务工程，改造提升10所乡镇卫生院，选派100名医生帮扶乡镇卫生院，培训3000名乡村医生，加强社区卫生服务建设，提升村卫生室服务能力；实施人口素质与优生促进工程，全面开展出生缺陷预防，提高出生人口素质。社会保障方面，实施武夷山新型农村社会养老保险试点；开工建设市级社会福利中心、1个县级社会福利中心、10所农村敬老院、50个社区居家养老服务中心站；实施特殊教育学校提升工程、“光明行动”1000例，资助贫困残疾人居家养护，实施残疾儿童康复项目；加快城市家庭保障性住房建设，完成廉租住房建设3.39万平方米，续建4.15万平方米，新开工1742套、9.64万平方米。强农惠农方面，实施水源保护、饮水安全、环境整治工程；深入开展家园清洁行动，80%的建制村实现垃圾简易处理；新建农村沼气池8000口；实施农村改厕项目；建设农村公路路网工程300公里，完成农村公路安保工程500公里，改造公路危桥60座，建设乡镇客运站5个；提高生态公益林补偿标准；调整完善政策性农业保险试点工作；积极做好库区移民工作；实施“造福工程”，力争搬迁10000人；加快省、市级扶贫开发重点村脱贫致富；实施“万村千乡市场工程”，建设300家农家示范店；建设农村社区综合维修服务体系；开展农村实用技术远程培训，培训农民4.5万人；推进新农村电气化县乡村建设；实施城乡通信信息化提升工程。文化建设方面，实施农民体育健身工程点180个；新建、改造9个乡镇综合文化站，建设25个乡镇文化信息资源共享点；实施农村电影“2131”放映工程；继续完善广播电视村村通、高山台站、县乡村光缆联网建设。和谐平安方面，深化平安创建活动；治理“餐桌污染”，建设“食品放心工程”；帮助驻延部队建设生产生活项目

全面推进生态市建设。加强水资源、土地资源、森林资源、矿产资源管理，巩固国家级生态示范区创建成果，全面推进生态市建设，鼓励创建国家级、省级环境优美乡镇、生态村。加快高速公路、高速铁路一重山绿色通道建设。严格执行木材加工企业准入制，全年木材砍伐量调减到180万立方米。科学调整树种结构，严厉打击和查处乱砍滥伐行为，发展生态茶果园，稳定森林覆盖率。进一步实施闽江流域水环境综合整治，治理工业污染、规模畜禽养殖污染和生活垃圾污水。加强节能减排工作，争取国家、省节能减排专项资金，实施一批节能、节水、节地、节材和资源综合利用项目，加快淘汰落后产能，发展绿色经济、循环经济和低碳经济，建设生态主导型区域经济。

维护社会和谐稳定。推进基层民主政治建设，切实维护人民群众的民主权利。加强公民思想道德建设，深入开展群众性精神文明创建活动。加强社区服务队伍建设，提高社区服务水平。加强社会治安综合治理，深入推进社会矛盾化解、社会管理创新、公正廉洁执法，努力提高维稳工作水平。规范信访秩序，完善信访工作长效机制。健全突发事件应急处置机制，有效预防和妥善处置群体性事件和突发公共事件。提升对甲型H1N1流感等重大疫病的防控能力。落实安全生产政府及部门的行政主体责任和企业的经济主体责任，有效防范重特大事故特别是交通安全事故的发生。继续做好援建彭州市九尺镇工作。支持工会、共青团、妇联等人民团体依照法律和各自章程开展工作。认真落实党的民族宗教政策。加强国防动员和民兵预备役工作，提高双拥共建水平。

三、强化责任，务求实效，增强政府执行力

一个干部最可贵的是责任心，一个团队最可贵的是执行力。各级政府和各部门必须按照科学发展观要求，切实加强自身建设，在推进闽北科学发展、跃升发展中体现更大作为。

（一）在解放思想中提升作为。围绕发展第一要务，加强理论学习，更新思想观念，转变思维方式，进一步增强闯拼意识，要有大胸怀，谋划大项目，争取大资金，推动大发展。认真研究海西政策、中央苏区政策、革命老区政策和中央扩大内需推动持续增长政策，以及省上产业振兴、惠民利民、小城镇发展、土地整理等政策，主动对接，敢于配套，跟踪落实，切实发挥政策的最大效应。立足于闽北发展阶段和发展水平，高起点、高标准、高水平编制“十二五”规划、主体功能区规划和专项规划。

（二）在加快发展中突出为民。把加快发展和改善民生有机结合起来，做到重大决策为民而定、政策举措应民而出、安危冷暖唯民所系，统筹更多的资源用在事关民生的大事、急事、难事上，让人民群众共享改革发展成果。始终带着责任、带着感情抓民生工程，落实各项惠民利民政策措施，细化工作方案，实行“阳光操作”，真正把实事办好。

（三）在转变职能中优化服务。用心服务基层、服务企业，努力建设服务型政府。积极稳妥地开展市县乡三级政府机构改革，深化行政审批制度改革，完善市、县两级行政服务中心，整合乡镇服务中心，规范市级招投标中心运作，成立信息服务中心，完善各类公开办事制度，推进网上审批和电子监察，增强公共服务能力。加强南平市行政学院建设和公务员培训，深化机关效能建设，加强绩效管理，优化创业竞赛考评机制，强化对重点工作定性定量的约束性要求，提高政府执行力。

（四）在依法履职中规范行政行为。依法接受人大及其常委会的法律监督和工作监督，主动接受政协民主监督，自觉接受社会各界和人民群众的监督，认真办理人大代表建议和政协提案，确保行政权力依法运行。健全依法科学民主决策制度，推进行政综合执法改革，规范行政自由裁量权，认真做好行政复议工作，完善执法过错追究制，维护人民群众合法权益，提高政府公信力。

（五）在从严治政中树好形象。深化政府系统反腐倡廉工作，注重从源头上防治腐败，着力建设科学严密完备管用的反腐倡廉体系。各级政府和各部门领导要切实履行“一岗双责”，落实主要负责人述职述廉制度。严格执行领导干部问责制，强化审计监督和行政监察，严肃查处建设规划、重大项目招投标、国有资产处置中出现的腐败行为，坚决纠正损害群众利益的不正之风，树立为民、务实、清廉的政府形象。

各位代表，海西绿色腹地建设进入新阶段，闽北发展面临新机遇。让我们高举中国特色社会主义伟大旗帜，紧密团结在以胡锦涛同志为总书记的党中央周围，深入贯彻落实科学发展观，在市委的正确领导下，团结和依靠全市人民，把握机遇，增强信心，拼搏奋进，乘势而上，为完成“十一五”规划各项任务、加快推进海西绿色腹地跃升发展而努力奋斗！
